# Application of polygene polymerization for insect-resistant poplar breeding

**DOI:** 10.48130/FR-2022-0003

**Published:** 2022-03-14

**Authors:** Yachao Ren, Yali Huang, Jun Zhang, Yan Dong, Jinmao Wang, Yanping Su, Minsheng Yang

**Affiliations:** 1 Forest Department, College of Forestry, Hebei Agricultural University, Baoding 071000, China; 2 Hebei Key Laboratory for Tree Genetic Resources and Forest Protection, Baoding 071000, China; 3 College of Life Science, Langfang Normal University, Langfang 065000, China

**Keywords:** Ecological security, Genetic transformation, Insect resistance, Polygene polymerization, Poplar, Unexpected effects

## Abstract

A major goal of poplar breeding is to obtain new insect-resistant poplar varieties through genetic engineering. However, engineering poplars with only a single insect-resistant gene has limitations, such as narrow insecticidal bands and the rapid development of insect tolerance to the gene. To expand insecticidal bands, delay the development of insect tolerance, and improve the insect resistance of transgenic poplars, polygene polymerization is increasingly being applied to cultivate insect-resistant poplars. In the present study, we discuss polygene polymerization, applications of the polygene combination strategy, the current state of research on transgenic poplar with multiple insect-resistant genes, including existing problems, and future research directions. We provide reference data to aid the cultivation and utilization of new polygenic insect-resistant poplar varieties.

## Introduction

Poplar is an economically important tree species in China that plays an important role in environmental protection, through wind mitigation and sand fixation. Intensive, large-scale poplar planting involves only a small number of poplar varieties and, due to the resulting low genetic diversity, poplar diseases and insect pests have become an increasingly serious issue. Poplar pests, such as *Hyphantria cunea* and *Anoplophora glabripennis*, spread easily and cause huge losses in forestry production, and also complicate environmental protection. Consequently, it is important to genetically engineer and cultivate novel insect-resistant poplar varieties^[1]^. The *Bacillus thuringiensis* insecticidal crystal protein gene (Bt gene) is currently the most widely used insect-resistant gene. Bt-gene-transgenic poplars have been used in small-scale field testing, pilot-scale production, and commercial applications, and have also been released into the environment. The introduction of the Bt gene improved the ability of poplars to target pests to some extent, but the transformation of a single insect-resistant gene has limitations, such as narrow insecticidal bands and rapid development of insect tolerance against the gene. To expand insecticidal bands, delay the generation of insect tolerance, and improve the insect resistance of transgenic poplars, the combined use of bivalent and multivalent genes has been introduced for the cultivation of insect-resistant poplars.

However, this approach might affect the expression efficiency of multiple foreign genes in transgenic plants, and the genes might also interact, resulting in variable improvements^[2−4]^. The random introduction of foreign genes and their expression products into recipient plants might have negative effects on the recipient plants themselves^[5,6]^. Furthermore, the expression products might also be transferred to non-target organisms through litter, root exudates, or the food chain^[7,8]^, which could further affect the ecosystem. Therefore, long-term monitoring of transgenic poplars is necessary. It is important to cultivate new transgenic poplar varieties with high insect resistance, unaffected growth, and improved environmental protection properties. In the present study, we discuss polygene polymerization, including its applications, and the current state of research. Furthermore, we discuss the application of polygenic polymerization for insect-resistant poplar breeding, problems with polygenic insect-resistant poplar, and research strategies and future directions. We provide reference data for the cultivation and utilization of new polygenic, insect-resistant poplar varieties.

## Polygene polymerization

A breakthrough in plant genetic engineering was the development of polygene polymerization, which enabled the aggregation of multiple desired plant traits instead of modification of only a single trait. At present, there are four main polygene polymerization methods: one-time transformation, multiple transformation, multi-vector co-transformation, and hybrid polymerization.

In one-time transformations, a single multi-gene vector is constructed and transformed only once. This method involves combining expression frames, polymerase hydrolysis, internal ribosome entry site (IRES) cleavage, and 2A cleavage. Combining expression frames connects the open reading frames (ORFs) of multiple target genes in series, and reconstructs them on a plant expression vector^[9]^. The target genes have their own regulatory elements, such as promoters and terminators, and can express their products independently. Polymerase hydrolysis fuses the sequences of multiple target genes, reconstructs them in the same ORF, transcribes them under the same promoter, and translates them to obtain a new multi-domain fusion protein formed by splicing multiple proteins with distinct functions. IRES is a special sequence of the 5' non-coding region of mRNA, which directly binds mRNA to ribosomes and initiates translation^[10]^. Two foreign target genes are connected by homologous recombination of the IRES and constructed on the same plant expression vector. Driven by the same promoter, the foreign target genes and IRES can be transcribed into the same mRNA, so that the target genes can be translated simultaneously. The self-cleaved 2A peptide, also known as cis-acting hydrolase element (CHYSEL), can form polyproteins during the translation of foreign genes^[11,12]^. IRES and 2A cleavage is more commonly applied in mammals than plants. Using the one-time transformation method, multiple target genes are generally integrated into a genetic site on the recipient chromosome. It is easy to obtain stable transgenic plants after transformation according to simple and convenient screening of their linked selective marker genes.

For multiple transformation, multiple plant expression vectors carrying one target gene each are constructed and used for multiple receptor transformations. This method can make full use of the existing mature vector construction and gene transformation technology. Without considering the capacity of the vector, the existing plasmid vector can meet the needs, and the number of target genes is not limited. However, because multiple independent transformation events are likely to integrate multiple target genes into multiple genetic loci, the separation of offspring of transgenic plants is complex and its stability is poor. It is difficult to obtain transgenic homozygous lines containing multiple target genes at the same time.

For multi-vector co-transformation, multiple genes located on different expression vectors are transferred into the same plant at the same time, which represents one-step transformation of multiple genes. Co-transformation methods include *Agrobacterium*-mediated transformation, particle bombardment, and PEG-mediated transformation; their efficiency varies. This method is simple and easy, but its co-integration frequency is unstable, which changes with different receptors, different transformation technologies and different structures, sizes and quantities of expression vectors. At the same time, there are great differences in the transformation rate of the target gene, and it is difficult to determine the integration site of the target gene in the recipient plant genome.

For hybrid polymerization, gene polymerization of transgenic single-gene or multi-gene plants is achieved through hybrid breeding^[9]^. This method can avoid the phenomenon that foreign genes are inserted into the same site and affect each other's expression in co-transformation. However, this method of breeding homozygous lines with different genes before hybridization is time-consuming and laborious, and is mostly suitable for existing transgenic homozygous lines. Considering the position effect of transgene and the stability of gene expression, it is usually necessary to expand the corresponding breeding population. Multivalent salt-tolerant and insect-resistant transgenic rice variants have been successfully cultivated by hybrid polymerization^[13,14]^.

Through the construction of multi-gene single vectors for genetic transformation, multi-gene plants can be obtained in one transgenic event. This method is simple and convenient, and thus widely used to transform plants, making it easy to obtain transgenic plants with stable inheritance of multiple traits.

## Application of polygene polymerization

Polygene polymerization advanced plant transgenic research. Wang et al.^[15]^ and Fan et al.^[16]^ simultaneously transformed bivalent genes for mannitol 1-phosphate dehydrogenase (*mtlD*) and sorbitol-6-phosphate dehydrogenase (*gutD*) into rice and 84K poplar through *Agrobacterium*-mediated transformation, and obtained transgenic rice and 84K poplar with strong salt tolerance. The polymerization of salt-tolerant genes improved the salt tolerance of recipient plants to some extent. Due to the huge losses in agriculture and forestry caused by pests and insect tolerance, combined application of insect-resistant genes in plant transgenic research is becoming more widespread.

Li et al.^[17]^ constructed the bivalent insect-resistant transgenic vector PCAM-Bt-CPTI, containing the Bt insecticidal gene *Cry1A(c)* and cowpea trypsin inhibitor gene *CPTI*, and genetically transformed the japonica rice line 'Zheda 19'. Insect resistance analysis showed that the three transgenic T1 generation lines were highly toxic to *Chilo suppressalis*^[17]^. Wu et al.^[18]^, Guo et al.^[19]^, and Li et al.^[20]^ also used Bt*Cry1A* and *CpTI* genes to obtain transgenic soybean and corn with bivalent insect resistance genes. Guo et al. transformed Bt and modified trypsin inhibitor *sck* genes to obtain a pure transgenic cotton line with significantly higher resistance to *Helicoverpa armigera* than that of a non-insect-resistant control and the insect-resistant control sGK321^[21]^. The combination of Bt and protease inhibitor genes can improve the insect resistance of plants to a certain extent.

In addition, combining multiple Bt genes is important for polygene polymerization. Lian et al. transformed tobacco with artificially modified *Cry1Ac* and *Cry1Ie* genes, and found that co-expression of the genes had a stronger insecticidal effect on *H. armigera*^[22]^. Cao et al. constructed separate *cry1C* and *cry1Ac* plant expression vectors and obtained transgenic Indian mustard with *cry1C* and *cry1Ac* bivalent insect-resistant genes through two transformations, which exerted a strong insecticidal effect on pests^[23]^. Yang et al. transformed *Cry1Ah* and *Cry1Ie* genes into corn, and molecular tests on the T0 and T1 generations confirmed that the two target genes were successfully integrated into the corn genome; moreover, the generations developed high resistance to *Ostrinia nubilalis*^[24]^. Polymerization of multiple insect-resistant genes plays an important role in improving the insect resistance of recipient plants and delaying the generation of tolerance in insects.

Insect resistance genes can also be combined with stress resistance genes or genes determining leaf color, which can improve multiple traits of recipient plants. Wang et al. introduced three foreign *Bacillus subtilis* genes, i.e., fructan sucrase (*SacB*), vitreoscilla hemoglobin (*vgb*), and bivalent stem borer resistance (Bt*Cry3A* + *OC-I*), as well as the regulatory gene *JERF36*, into *Populus* × *euramericacana* 'Guariento' via particle bombardment^[25]^. They demonstrated that the foreign gene was integrated into the poplar genome, and that the Bt*Cry3A* gene was expressed. Xiong et al. transformed alfalfa cotyledons with multi-gene plant expression vectors carrying the insect-resistant gene *Cry1Aa*/*Cry1Ac*, *Pinellia ternata* lectin gene *pta*, and herbicide-resistant gene *bar*^[26]^. After continuous herbicide screening, transgenic plants that expressed foreign genes were obtained. Zhou constructed a polymeric expression vector carrying the insect-resistant gene Bt*cryII* and leaf color gene *CmOr* based on IRES elements, transformed *Populus tomentosa* TC1521, and obtained seven transgenic lines integrating Bt*cryII* and *CmOr* genes into their genome^[27]^. The relative expression levels were significantly higher than that of wild-type plants. In conclusion, polygene genetic transformation plays an important role in plant breeding.

## State of research on transgenic poplar with multiple insect-resistant genes

Poplar is an economically important tree species in China. Due to intensive, large-scale poplar planting, poplar diseases and insect pests have become an increasingly serious issue. Through the transfer of a single insect-resistant gene, the insect resistance of poplars could be improved to some extent. However, large-scale planting of transgenic poplar with a single transferred insect-resistant gene causes target insects to develop tolerance against the insect-resistant gene over time. Therefore, polygene polymerization plays a particularly significant role in insect-resistant poplar breeding. Polymerizing two or more insect-resistant genes with different insecticidal mechanisms is the most effective way to prevent pests from generating tolerance, thus improving the insect resistance of transgenic plants.

## Combination of bivalent and multivalent insect resistance genes

In recent years, a variety of insect-resistant genes have been studied and transformed, including the Bt gene and genes for arrowhead protease inhibitor *API*, cowpea trypsin inhibitor *CpTI*, potato protease inhibitor *PI*, rice sulfhydryl protease inhibitor *OC-I*, *Pinellia ternata* lectin *pta*, and spider insecticidal peptide. Combining bivalent and multivalent insect-resistant genes advanced the cultivation of new insect-resistant poplar varieties.

Cornu et al. transformed the genes Bt*Cry3A* and *PI* into poplar, and obtained bivalent insect-resistant poplar^[28]^. The transgenic plants prevented larval growth, inhibited pest development, and improved larval mortality. Tian et al. transformed a partially modified Bt*Cry1Ac* gene and the *API* gene into poplar 741^[29]^. They obtained transgenic plants with high resistance to lepidopteran pests such as *H. cunea*, *Clostera anachoreta*, and *Lymantria dispar*, which delayed the development of resistance of pests and expanded the insecticidal spectrum. Their plants were the first transgenic poplars with double insect-resistant genes obtained in China. Following the transformation of poplar 741 with bivalent insect-resistant genes, further poplar clones with multiple insect-resistant genes were transformed (Table 1). Wang et al. transformed Bt*Cry1Ac* and *API* genes into *Populus cathayensis* Rehd and obtained transgenic plants that were further screened using antibiotics^[30]^. Yang et al. transformed triploid *Populus tomentosa* with the genes Bt*Cry1Ac* and *API*, and fed the leaves of 28 obtained transgenic lines to *L. dispar* and *C. anachoreta* larvae^[31]^. They showed that 39.3% of the transgenic lines had a larvae mortality rate of over 80%, and 25% of the lines had a mortality rate of 60%–80%. Furthermore, the highly resistant lines showed inhibitory effects on the growth and development of surviving larvae. Li et al. transformed *Populus tomentosa* clone 85 with the genes Bt*Cry1Ac* and *API*, and conducted similar larvae mortality experiments^[32]^. They showed that the *L. dispar* larvae mortality rate of transgenic plants reached 60.0%–77.8%, and the growth and development of surviving larvae were significantly inhibited. Yang et al. also transformed *Populus euramericana* 'Neva' with the genes Bt*Cry1Ac* and *API*, proved that the target gene had been integrated into the genome of *P. euramericana* 'Neva', and obtained high-quality transgenic lines with normal growth and strong insect resistance^[33]^. In conclusion, combining the genes Bt and *API* for poplar transformations has led to multiple successful and promising transformations.

**Table 1:** Transgenic poplars with bivalent or multivalent insect-resistant genes cultivated in China.

Gene	Poplar varieties	Author	Year
Bivalent or multivalent insect-resistant genes			
*BtCry1Ac* + *API*	Poplar 741	Tian et al.^[29]^	2000
	*Populus cathayana* Rehd.	Wang et al.^[30]^	2003
	*P. tomentosa*	Yang et al.^[31]^	2006
	*Populus tomentosa* clone 85	Li et al.^[32]^	2007
	*P. euramericana* 'Neva'	Yang et al.^[33]^	2012
*BtCry1A* + *CpTI*	*P. deltoides* Marsh. × *P. simonii* Carr.	Rao et al.^[8]^	2001
*OC-I* + *BtCry3A*	*Populus alba* × *P. glandulosa* cv '84k'	Zhang et al.^[34]^	2005
*Cry1A(b)* + spider insecticidal peptide gene	*Populus simonii* × *P. nigra*	Jiang et al.^[35]^	2004
	*Populus davidiana* Dode × *P. bollena* Lauche	Zuo et al.^[36]^	2009
	*P. nigra* × *P. deltoids* "108"	Zou et al.^[37]^	2010
	(*P. simonii* × *P. nigra*) × P.15ACL	Song^[38]^	2010
*Cry3A* + *Cry1Ac* + *API*	Poplar 741	Wang et al.^[39]^	2012
*Cry1Ac* + *Cry3A*	*P. deltoides* cv. "Juba"	Dong et al.^[2]^	2015
*Cry3A* + *Cry1Ac*	*P. euramericana* 'Neva'	Ren et al.^[40]^	2021
Combination of double insect-resistant genes and other resistance genes			
*BtCry3A* + *OC-I* + *Vgb* + *SacB* + *JERF36*	*P. euramericana* 'Guariento'	Wang et al.^[25]^	2006
*Cry1Ac* + *Cry3A* + *BADH*	*P. euramericana* 'Neva'	Ren^[41]^	2013
*Cry1Ac* + *Cry3A* + *NTHK1*	*P. euramericana* 'Neva'	Zhang^[42]^	2013
*Cry3A* + *Cry1Ac + mtlD* + *BADH*	*P. nigra* L.	Zhou et al.^[43]^	2020

Other studies focused on the combination of the Bt gene and other insect-resistant genes. Rao et al. transformed a modified Bt*Cry1A* gene and the *CpTI* gene into poplar NL-80106, and obtained a transgenic bivalent insect-resistant line with insecticidal activity against first instar *L. dispar* larvae^[8]^. Zhang et al. transformed *OC-I* and Bt*Cry3A* genes into *Populus alba* × *P. glandulosa* cv '84k', and proved that the exogenous genes were integrated into the genome of 13 transgenic *P. alba* × *P. glandulosa* cv '84k' lines^[34]^. Feeding tests revealed differences in the lethality of the transgenic lines on *Anoplophora glabripennis* larvae. Line BOGA-39 had a lethality rate of more than 40% and inhibited the growth of surviving larvae, while the lethality of other lines was low. Jiang et al. formed a fusion gene consisting of the genes for Bt C peptide and spider insecticidal peptide, introduced it into *Populus simonii*, and obtained a transgenic line with high insect resistance^[35]^. Zuo et al. transformed the Bt gene and gene for spider insecticidal peptide into *Populus davidiana* Dode × *P. bollena* Lauche, and proved that the foreign gene was integrated into the genome^[36]^. Zou et al. tested the insect resistance of two *Populus nigra* × *P. deltoids* '108' lines against *L. dispar* larvae, and showed that the development of the first to fourth instar larvae feeding on the two lines took 3.28 and 3.6 days longer than that of larvae feeding on the control, respectively^[37]^. Song transformed a chimeric gene consisting of the genes for spider insecticidal peptide and Bt toxin protein into (*P. simonii* × *P. nigra*) × P.15ACL No.1 via *Agrobacterium*-mediated transformation, and proved that the target gene had been integrated into the poplar genome of three lines^[38]^. The combination of the Bt gene and other insect-resistant genes improved the insect resistance of poplar to a certain extent.

Further studies focused on the combination of double Bt genes. Wang et al. introduced the *Cry3A* gene into poplar 741 lines that were previously transformed with *Cry1Ac* and *API* genes, and obtained poplar 741 with double Bt genes^[39]^. Cry1Ac and Cry3A insecticidal proteins were expressed in the transgenic lines. And the transgenic lines had double resistance to *Plagiodera versicolora* and *H. cunea*. Dong et al. used two plant expression vectors, pCAMBIA1305-Cry1Ac-Cry3A and p71A68Y71, carrying the double Bt genes *Cry1Ac* and *Cry3A* to transform *P. deltoides* cv. 'Juba'^[2]^. They proved that the exogenous Bt genes were integrated into the genome of *P. deltoides* cv. 'Juba', and were transcribed and translated. The double Bt transgenic lines showed no resistance to *H. cunea*, but high resistance to the first to third instar larvae of *P. versicolora*. Ren et al. introduced the double Bt genes *Cry3A* and *Cry1Ac* into the same plant expression vector, transformed *P. euramericana* 'Neva', and obtained transgenic lines with high insect resistance to *H. cunea*, *Micromelalopha troglodyta* and *P. versicolora*, as well as inhibitory effects on the growth and development of *A. glabripennis* larvae^[40]^. The generation of different types of transgenic poplars with multiple insect-resistant genes provides new germplasm resources for the cultivation of new poplar insect-resistant varieties.

## Combination of multiple insect-resistant genes and other resistance genes

Combinations of multiple insect-resistant genes and other resistance genes are increasingly being transformed into poplar to obtain lines with multiple improved traits (Table 1). Ren^[41]^ and Zhang^[42]^ combined the double Bt gene *Cry1Ac*+*Cry3A* with the salt-tolerant genes *BADH* and *NTHK1*, respectively, to transform *P. euramericana* 'Neva'. Yang et al.^[43]^ and Liu et al.^[44]^ conducted in-depth studies on the two *P. euramericana* 'Neva' lines generated by Ren and Zhang, and detected toxic protein expression in all transgenic lines via ELISA. Furthermore, they found that the transgenic lines showed intermediate resistance to target insects, and that the lines' salt tolerance was improved, although the insecticidal effect on lepidopteran target insects was weak. Zhou et al. transformed the four foreign genes *Cry3A*, *Cry1Ac*, *mtlD*, and *BADH* into *Populus nigra* L., and obtained transgenic lines with high insect resistance and intermediate salt tolerance^[45]^. These studies demonstrate that the transfer of multiple genes can improve multiple plant traits to a certain extent, and that the generation of transgenic plants enables the cultivation of new varieties.

## Administrative examination and approval of transgenic poplar with multiple insect-resistant genes in China

After more than 20 years of research in China, major advances have been made in the fields of insecticidal gene transfer, toxin expression and transportation, polygene polymerization, biosafety, and insect resistance sustainability and stability. The State Forestry and Grassland Administration established several regulatory frameworks for the genetic engineering of forests. All research on insect-resistant trees must proceed according to certain stages, including project application and approval, small-scale field testing, environmental release, pilot-scale production, and commercialization; moreover, each stage must be evaluated by experts^[46]^. Some transgenic poplars with multiple insect-resistant genes have entered the stages of small-scale field testing, environmental release, pilot-scale production, and commercialization (Table 2). In 2002, 'Transgenic poplar 741 with double insect-resistant genes' obtained a licence for the commercialization of transgenic trees, a certificate for new plant varieties, and a certificate for improved trees. Other transgenic poplars that entered the small-scale field testing stage include transgenic *Cry3A* + *OC-I* + *vgb* + *SacB* + *JERF36* multi-insect-resistant gene *P. euramericacana* 'Guariento', transgenic *Cry1A(b)* + spider insecticidal peptide *P. simonii*, transgenic *Cry1Ac* + *API* *P. tomentosa* Carr. clone BL73, transgenic *Cry3A* + *Cry1Ac* + *API* poplar 741, transgenic *Cry1Ac* + *API* *P. euramericana* 'Neva', transgenic *Cry3A* + *Cry1Ac* + *BADH* *P. euramericana* 'Neva', transgenic *Cry3A* + *Cry1Ac* + *NTHK1* *P. euramericana* 'Neva', transgenic *Cry3A* + *Cry1Ac P. euramericana* 'Neva', and *P. deltocdes* '55/56' × *P. deltocdes* '2KEN8'. Among those, transgenic *Cry3A* + *OC-I* + *vgb* + *SacB* + *JERF36* *P. euramericacana* 'Guariento', transgenic *Cry1Ac* + *API* *P. euramericana* 'Neva', transgenic *Cry3A* + *Cry1Ac* + *BADH* *P. euramericana* 'Neva', and transgenic *Cry3A* + *Cry1Ac* + *NTHK1* *P. euramericana* 'Neva' entered the environmental release and pilot-scale production stage. Poplar is a perennial tree with a long growth cycle. It is necessary to carry out long-term ecological risk assessments before beginning field experiments and commercial production of poplar. The approval requirements for new varieties are high in China; consequently, although further insect-resistant poplar varieties have been obtained, no new commercial varieties have yet been approved.

**Table 2:** The administrative approval of transgenic multiple insect-resistant genes poplars in China.

Gene	Poplar varieties	Small-scale field testing	Environmental release and pilot-scale production	Commercialization
*Cry1Ac* + *API*	Poplar 741	1996–1998	1999–2001	2002–2007
*Cry3A* + *OC-I* + *Vgb* + *SacB* + *JERF36*	*P. euramericana* 'Guariento'	2005–2008	2009–2013	
*Cry1A(b)* + spider insecticidal peptide gene	*P. simonii* × *P. nigra*	2006–2010		
*Cry1Ac* + *API*	*P. tomentosa* Carr. Clone BL73	2011–2014		
*Cry3A* + *Cry1Ac* + *API*	Poplar 741	2011–2014		
*Cry1Ac* + *API*	*P. euramericana* 'Neva'	2011–2014	2016–2018	
*Cry3A* + *Cry1Ac* + *BADH*	*P. euramericana* 'Neva'	2016–2018	2019–2021	
*Cry3A* + *Cry1Ac* + *NTHK1*	*P. euramericana* 'Neva'	2016–2018	2019–2021	
*Cry3A* + *Cry1Ac*	*P. euramericana* 'Neva'	2019–2021		
*Cry3A* + *Cry1Ac*	*Populus deltocdes* '55/56' × *P. deltocdes* '2KEN8'	2019–2021		

## Existing problems and perspectives

### Expression efficiency of combined genes after polygene polymerization

After polygene polymerization, many traits of receptor plants can be improved to a certain extent, but the expression efficiency of combined genes may differ. The *Cry3A* gene was introduced into poplar 741 line pB29 with *Cry1Ac* + *API* genes via secondary transformation, and transgenic three-gene (*Cry3A* + *Cry1Ac* + *API*) poplar 741 lines were obtained. Most lines had high insect resistance to target insects, while the insect resistance of some lines to the fourth instar larvae of *H. cunea* was significantly reduced^[39,47]^. These studies showed that the two Bt insect-resistant genes in transgenic three-gene poplar 741 can be highly expressed; however, random insertion of the *Cry3A* gene can reduce the expression of the *Cry1Ac* gene in the original poplar 741 line pB29.

Similarly, some studies have shown that after a single vector of bivalent or multivalent genes was transformed into a plant, the insertion positions of the foreign genes in the receptor genome were the same, but their expression levels were different^[2−4]^. Dong et al. constructed the genes *Cry3A* and *Cry1Ac* on an expression vector and transformed them into *P. deltoides* cv. 'Juba'^[2]^. They found that the transgenic lines had no resistance to *H. cunea*, but high resistance to *P. versicolora.* This might not only be related to the genes and promoter types themselves, but also to the interaction between genes and the structure of vectors. Xu et al. compared the expression of foreign genes in four double Bt gene vectors, and studied the effect of vector structure on the expression of double Bt genes^[48]^. They found that the structure of the nuclear matrix binding region in the vector improved the expression levels and stability of Bt genes. Different promoter and enhancer sequences can improve the expression level of Bt genes, and when the genes are upstream of T-DNA, their expression levels are lower. Qiu et al. compared the expression levels and insect resistance of Bt genes in five double Bt gene vector tobacco lines, and found that the arrangement sequence and direction of the target gene expression cassette in T-DNA have a significant impact on the expression of Bt genes^[49]^. When the expression cassette of the two target genes was reversely arranged, their expression was promoted. These studies provide a reference for improving the expression of genes in multi-gene vectors and clarifying the possible interaction between multiple genes, and they also provide a theoretical foundation for polygene polymerization for transgenic plants.

### Effects of polygene polymerization on plant receptors

Polygene polymerization not only improves target traits of recipient plants, but can also affect their cellular mechanisms and metabolism. The insertion of foreign genes into the receptor genome often destroys the structure of the original nucleotide sequence at the insertion site, interferes with gene transcription, and even causes gene silencing. In addition, the foreign gene product itself might also affect the transcription and expression of original genes, thereby changing the metabolism of the recipient plant and generating unexpected, unpredictable effects^[5,6,50]^. The insertion and expression of foreign genes in transgenic plants might affect their phenotypic characteristics, nutritional components, and secondary metabolites^[51]^.

Known phenotypic variation traits in transgenic *Arabidopsis thaliana*, rice, and tomato include plant dwarfing, super-height, leaf shrinkage, deformity, and leaf color changes^[52−55]^. These phenotypic variations might be caused by the over-expression of foreign genes, gene silencing caused by interference inhibition, and activation or inactivation of other genes caused by the insertion of T-DNA^[56,57]^. Studies on the T-DNA insertion positions of transgenic rice with the insect-resistant gene *Cry1Ca* and herbicide gene *bar* found that the insertion of T-DNA led to changes in the expression of upstream and downstream genes, which might in turn lead to unexpected effects such as shorter plant height and a low seed setting rate^[58]^. Zhou et al. analyzed the metabolic characteristics of transgenic rice with insect-resistant *cryIAc* and *sck* genes, and wild rice, and found that sucrose, mannitol, and glutamic acid levels increased significantly in transgenic compared to wild rice^[59]^. Huang et al. found that the height and diameter at breast height of the transgenic poplar 741 line Pb29 with *Cry1Ac* and *API* genes were smaller than those of the control poplar 741 line^[60]^. However, the molecular mechanism underlying these unexpected effects is still unclear. Potential causes include host gene destruction or DNA sequence rearrangement at the insertion site of foreign genes^[61]^, somatic variation during the construction of transgenic strains^[62,63]^, excessive biomass and energy consumption of host cells during foreign gene expression^[64]^, and interactions between exogenous and endogenous proteins^[65]^. However, there is no direct empirical evidence to support these hypotheses. With the advancement of multi-omics technology, differences in transcription, protein, and metabolite levels between transgenic plants and non-transgenic controls can be identified through transcriptome, proteome and metabolome analysis. By applying molecular techniques such as yeast two-hybrid screening, bimolecular fluorescence complementation, and co-immunoprecipitation, the function of endogenous proteins interacting with foreign proteins can be explored to determine the molecular mechanisms underlying phenotypic changes in transgenic plants, and to provide references for safety assessments and appropriate utilization of transgenic plants.

### Ecological safety of polygene transgenic poplar

After the commercialization of the two transgenic poplar varieties Bt gene *P. nigra* and double insect-resistant genes poplar 741, Chinese researchers have generated many other insect-resistant poplar varieties. However, considering the possible impact of transgenic poplars on ecosystems, new commercial varieties have not yet been approved. There are also widespread national and international concerns about possible ecological security problems caused by transgenic poplar, such as the spread of the bacterium *Agrobacterium tumefaciens* used in transformations, the stability of foreign genes, the resistance of target organisms, the impact of foreign genes on biodiversity, and horizontal gene transfer^[66]^.

### Survival and spread of *Agrobacterium tumefaciens* and horizontal transfer of foreign genes

*Agrobacterium*-mediated transformation is currently the most widely used plant transformation method. After the transformed plant is obtained, residual *A.* *tumefaciens* bacteria in the plant not only affect the survival of the transformed plant^[67]^ and reliability of PCR results^[68]^ but also causes the drift of foreign genes^[69]^. In one study, residual *A.* *tumefaciens* in triploid *P. tomentosa* transformed with Bt*Cry1Ac* + *API* genes by *Agrobacterium*-mediated transformation were monitored, and after 28 transgenic lines were cultured in tissue culture flasks for 24 months, residual *A. tumefaciens* were detected in three lines. The tissue culture seedlings of the three lines carrying *A. tumefaciens* bacteria were transplanted into flowerpots. After 1 month of indoor culturing, *A. tumefaciens* was detected in the rhizosphere soil of one line^[69]^. This result shows that *A. tumefaciens* can carry the target gene into the environment. Hou performed horizontal transfer detection of foreign genes in soil microorganisms, peripheral plants, and insects sampled from experimental sites of *P. euramericacana* 'Guariento' and *P. alba* × *P. glandulosa* with multi-genes^[70]^. The target gene and its marker gene *NPTII* were not detected in the samples, which indicated that the likelihood of horizontal transfer of foreign genes from transgenic poplar is extremely low within 2 years of planting. Zhu et al. performed target gene detection via PCR of mixed samples of weeds growing within an 8-year-old multi-gene *P. euramericacana* 'Guariento' forest, and on DNA samples from soil microorganisms. No target gene was detected, indicating that there was no horizontal transfer of foreign genes from multi-gene *P. euramericacana* 'Guariento'^[71]^. Li et al. studied the influence of the Bt-spider neurotoxic peptide recombinant gene from *P. simonii* on the soil microbial community structure, and detected no target gene in the rhizosphere of 1-month-old transgenic plants. However, they found several target-gene-positive bacteria in the rhizosphere of 7-month-old plants, and the proportion of positive bacteria colonies was 2%–5%^[72]^. This indicated that foreign genes may have been transferred horizontally. Therefore, to effectively prevent horizontal transfer of target genes and the spread of *A. tumefaciens* in the environment, strict screening before transplanting transgenic poplars is needed, as well as continuous monitoring of transgenic poplars and soil microorganisms in the field.

### Impact on target and non-target insects

Poplar has a long growth cycle. Leaf-eating and piercing-sucking pest insects can directly contract insecticidal proteins in transgenic Bt poplar during their growth process, and might indirectly and continuously transmit them to pest predator insects, thus affecting the entire insect community^[8]^. Most studies assessing the safety of genetically modified crops showed that transgenic lines had no negative impact on the ecosystem^[73−75]^. However, some studies showed that target pests develop resistance to Cry1Ac proteins^[76−79]^, and that Bt-transformed plants can promote growth of the population of non-target pests and inhibit that of pest predator insects^[80,81]^.

Zhang et al. found that the transgenic poplars with Bt*-Cry3A* and oryzacystatin I bivalent genes maintained their resistance to Coleoptera in the field^[82]^. Hou studied the effects of *vgb*, *SacB*, Bt*Cry3A*+*OC-1*, *JERF36* multi-gene *P. euramericacana* 'Guariento' and Bt*Cry3A*+*OC-1* transgenic *P. alba* × *P. glandulosa* on target and non-target insects, and found that the number of target insects in a transgenic *P. euramericacana* 'Guariento' forest, and the abundance of Coleoptera in a transgenic *P. alba* × *P. glandulosa* forest, were generally lower than that of a non-transgenic control forest, demonstrating insect resistance of the transgenic forests^[70]^. In terms of the abundance of non-target insects, there was no significant difference between the transgenic *P. euramericacana* 'Guariento' forest and control forest. However, individual lines reduced the abundance of target insects while increasing the abundance of non-target insects. The abundance of pest predator insects in the transgenic multi-gene *P. euramericacana* 'Guariento' and *P. alba* × *P. glandulosa* forests was higher than that of control forests; therefore, transgenic poplar positively impacted pest predator insects, consistent with results for insect-resistant gene poplar 741^[83]^. Huang et al. found in a 6-year-long study that the poplar 741 line with *Cry1Ac* + *API* genes can significantly inhibit lepidopteran, coleopteran, and other pests, without affecting non-target insects^[60]^. Previous studies have shown that transgenic poplar 741 with double insect resistance genes does not cause an increase in the abundance of non-target pests^[84]^; however, Guo et al. found that transgenic *Cry1Ac* + *API* *P. euramericana* 'Neva' not only inhibits lepidopteran pests, but also causes a significant increase in the abundance of piercing-sucking insects^[85]^. Therefore, while adverse effects of transgenic multi-gene poplar on pest predator insects are not supported by multiple studies, due to the unpredictable impact of transgenic poplar on the environment, long-term follow-up field investigations are needed to provide data for safety evaluation systems and commercialization of transgenic poplar.

### Impact on soil microbial ecosystem

Proteins released by transgenic plants can enter the soil through pollen, litter, or root exudates^[7]^. An increasing number of studies are focusing on the effects of transgenic poplars on soil microbial ecosystems.

When investigating soil microorganisms in the rhizosphere of transgenic multi-gene *P. euramericacana* 'Guariento', no significant difference in the number of soil microorganisms was found between transgenic and non-transgenic lines, indicating that transgenic multi-gene *P. euramericacana* 'Guariento' did not affect the number of soil microorganisms at the experimental site^[70]^. Four lines of transgenic Bt + *CpTI* Nanlin 895 poplar and a control line were analyzed for culturable groups of rhizosphere soil microorganisms, and it was found that the number of bacteria, fungi, and Actinomycetes among rhizosphere soil microorganisms of transgenic poplar was not significantly different from that of the control^[86]^. Zhen et al.^[87]^ and Li et al.^[88]^ investigated changes of soil microbial communities during the growth of transgenic double insect-resistant genes poplar 741 and transgenic multi-gene *P. euramericacana* 'Guariento', respectively, over 4 years and found that transgenic multi-gene poplar had no significant effect on the number of rhizosphere soil microorganisms. Lv found that there were differences in the number of rhizosphere soil Bacilli, Actinomycetes, nitrogen-fixing bacteria, and molds between the ABJ series of transgenic *JERF36 *+ *vgb *+ Bt*Cry3A *+ *OC-1* *P. alba* × *P. Berolinensis* and a control group, but none of them reached statistical significance (*p* > 0.05)^[89]^. In another study, the number of bacteria, fungi, and Actinomycetes in the soil of an 8-year-old transgenic multi-gene *P. euramericacana* 'Guariento' forest was studied using the dilution plate method^[71]^. The study showed that the number of soil microorganisms was not significantly affected. When analyzing the carbon source utilization ability, richness index, dominance index, and evenness index of soil microorganisms in the rhizosphere of transgenic multi-gene *P. euramericacana* 'Guariento', a non-transgenic control, and planting soil, it was found that the transgenic multi-gene *P. euramericacana* 'Guariento' did not affect the soil microbial community^[90]^. In conclusion, current research indicates that transgenic poplar has no significant effect on the soil microbial ecosystem. However, due to the long growth cycle of trees, the long-term expression of foreign genes in transgenic plants requires continuous monitoring to exclude long-term effects on the soil microbial ecosystem.

### Safe utilization of transgenic poplar

In China, transgenic poplar planting has effectively inhibited the rapid spread of target pests and significantly reduced the amount of required pesticide^[91]^. However, whether transgenic multi-gene poplar affects the ecosystem is still being assessed, and safe utilization of transgenic multi-gene poplar is still of high importance. Large-scale, long-term planting of transgenic plants might cause insects to generate resistance against transgenic trees; to prevent resistance generation, multiple strategies can be adopted, such as the genetic strategy, gene promoter strategy, gene expression strategy, refuge strategy, genetic diversity protection strategy, and barrier protection effect^[92,93]^. In addition, transgenic poplars can be utilized in agroforestry systems to avoid the risks associated with large-scale planting^[94]^. To prevent transgenic plants from causing adverse effects on human health and the environment, developed countries have successively established biosafety management and evaluation systems^[95]^. China have also implemented management measures for genetically modified organisms, and standardized the examination and approval of genetically modified forest engineering activities. The Chinese State Forestry Administration is responsible for the examination and approval of genetically modified forest engineering activities, and China implemented regulations on biosafety monitoring and management of genetically modified trees, including strict monitoring of the growth of these trees and possible problems. When establishing, improving, and implementing regulations and systems for transgenic tree management, safer methods to obtain transgenic poplars from target genes, and safer genetic engineering technologies, should be further explored.
